# TRIM27 Ubiquitinates and Degrades PPARγ to Promote Osteogenic Differentiation of BMSCs and Alleviate Osteoporosis

**DOI:** 10.1155/ije/6777760

**Published:** 2026-06-08

**Authors:** Liya Zhang, Junfeng Li, Benjuan Wu

**Affiliations:** ^1^ Department of General Medicine, Tianjin First Central Hospital, Tianjin, 300192, China, tj-fch.com; ^2^ Department of Rheumatology and Immunology, Tianjin First Central Hospital, Tianjin, 300192, China, tj-fch.com

**Keywords:** BMSCs, osteoporosis, PPARγ, TRIM27

## Abstract

**Objective:**

This study elucidates the role of TRIM27 in osteogenic differentiation and osteoporosis pathogenesis, focusing on its regulatory mechanism through PPARγ.

**Methods:**

TRIM27 expression was assessed during osteogenic differentiation of bone marrow mesenchymal stem cells (BMSCs) via RT‐qPCR and western blot. TRIM27 was knocked down (sh‐TRIM27) or overexpressed (Lv‐TRIM27) to evaluate its impact on alkaline phosphatase (ALP) activity, mineralization (Alizarin Red S staining), and osteogenic markers (COL1A1, OPN, and OCN). An ovariectomized (OVX) mouse osteoporosis model was established to analyze TRIM27 expression and bone microstructure (H&E staining). Co‐immunoprecipitation (Co‐IP) and cycloheximide chase assays identified TRIM27–PPARγ interactions and degradation dynamics. Rescue experiments combined sh‐TRIM27 with PPARγ knockdown (sh‐PPARγ) or the agonist rosiglitazone (ROZ).

**Results:**

TRIM27 expression increased 2.1‐fold during BMSC osteogenic differentiation but decreased in OVX mice (*p* < 0.01 vs. sham). TRIM27 overexpression enhanced ALP activity, mineralization, and osteogenic markers (*p* < 0.05), while knockdown exerted opposite effects. TRIM27 directly bound PPARγ and promoted its ubiquitin‐mediated degradation. PPARγ knockdown reversed sh‐TRIM27‐mediated inhibition of osteogenesis. In OVX mice, Lv‐TRIM27 reduced bone loss by suppressing PPARγ, whereas ROZ abolished this protective effect.

**Conclusion:**

TRIM27, as a key osteogenic promoter, ameliorates osteoporosis by targeting the degradation of PPARγ. Our research reveals the potential of TRIM27 in treating bone metabolism disorders and may become a potential target for the treatment of osteoporosis.

## 1. Introduction

Osteoporosis has emerged as a critical global public health challenge, characterized by increased fracture risks that significantly impair patients’ quality of life and elevate mortality rates [1]. Epidemiological data indicated that this metabolic bone disorder affects approximately 200 million individuals worldwide, with particularly high prevalence among elderly populations [2]. The pathogenesis involves disrupted bone homeostasis, marked by accelerated bone resorption and compromised structural integrity. Central to this pathological progression is the lineage commitment imbalance in bone marrow mesenchymal stem cells (BMSCs), where aberrant activation of adipogenic differentiation coincides with progressive attenuation of osteogenic potential [3]. Mechanistic studies [4, 5] revealed that excessive adipocyte formation not only competitively inhibits osteoblast differentiation but also exacerbates bone microenvironment dysregulation through adipokine‐mediated metabolic disturbances. Therefore, a comprehensive understanding of the molecular regulatory mechanism of osteogenic differentiation of BMSCs will provide key evidence for achieving targeted therapeutic strategies for osteoporosis.

BMSCs directly counteract the pathological progression of osteoporosis through osteoblast differentiation, replenishing depleted osteogenic precursors and restoring the balance between bone formation and resorption [6, 7]. The osteogenic capacity of BMSCs is orchestrated through a complex molecular network. Age‐related dysregulation of the WNT/β‐catenin pathway emerges as a critical pathogenic mechanism, wherein FKBP5 upregulation promotes β‐catenin ubiquitination, thereby suppressing osteoblastogenesis [3]. Pharmacological inhibition of FKBP5 with compounds such as SAFit2 rescues bone mineral density (BMD) in animal models, validating this axis as a druggable target. Concurrently, mitochondrial dysfunction associated with SIRT3 deficiency accelerates BMSC senescence and impairs differentiation, underscoring the role of metabolic reprogramming in osteoporosis pathogenesis [8]. The pathophysiology of postmenopausal osteoporosis involves heightened BMSC apoptosis mediated by the FoxO1/NF‐κB signaling axis, which natural compounds such as *Eucommia ulmoides* flavonoids mitigate by enhancing FoxO1 nuclear translocation and antiapoptotic gene expression. Consequently, synergistic therapeutic strategies combining BMSC‐based approaches, extracellular vesicles, and traditional Chinese medicine formulations represent a promising avenue for osteoporosis treatment [9].

Tripartite motif‐containing (TRIM) 27, a member of the TRIM protein family, functions as a critical regulator across diverse physiological and pathological contexts [10]. As an E3 ubiquitin ligase, it mediates protein degradation via ubiquitination and modulates key signaling networks including Wnt/β‐catenin and PI3K/AKT pathways, thereby influencing cellular processes such as proliferation, differentiation, and apoptosis [11]. In colorectal cancer, TRIM27 overexpression correlates with poor prognosis, driving epithelial–mesenchymal transition through activation of p‐AKT signaling. Similarly, in esophageal squamous cell carcinoma, it enhances glycolysis and suppresses apoptosis via PTEN/AKT pathway modulation. Yao et al. reported [12] that downregulation of TRIM27 leads to the inhibition of BIRC5 through the upregulation of LATS2 and the inhibition of Yes‐associated protein 1 (YAP1), thereby suppressing the development of gastric cancer. In osteoclastogenesis, TRIM27 acts as a negative regulator by interacting with TAB2, a pivotal adaptor protein in the NF‐κB signaling cascade [13]. This interaction promotes lysosomal degradation of TAB2, thereby inhibiting TRAF6 autoubiquitination and suppressing RANKL‐induced NF‐κB activation. Despite these advances, the role of TRIM27 in osteoporosis pathogenesis, particularly its impact on BMSC differentiation and molecular regulation, remains unclear.

## 2. Materials and Methods

### 2.1. Cell Culture

Following anesthesia, bone marrow was flushed from the tibiae and femora of 8‐week‐old mice using α‐minimal essential medium (α‐MEM) to isolate primary BMSCs. The isolated cells were seeded in 90‐mm culture dishes and maintained for 16 h. Adherent cells were subsequently harvested for BMSC culture. Osteogenic differentiation was induced using a medium supplemented with 10 mM β‐glycerophosphate, 10 nM dexamethasone, and 50 μg/mL ascorbic acid.

### 2.2. Ovariectomy Mice Model

Animal experiments were approved by the Animal Ethics Committee of Tianjin First Central Hospital. A total of 24 female mice were randomly divided into four groups (*n* = 6 per group): sham group, ovariectomized (OVX) group, OVX + Lv‐TRIM27 group, and OVX + Lv‐TRIM27 + rosiglitazone (ROZ) group. Mice in the sham group underwent exposure of the ovaries without removal, while mice in the other groups underwent bilateral ovariectomy (OVX) as previously described [14]. OVX + Lv‐TRIM27: Lentivirus overexpressing TRIM27 (Lv‐TRIM27) was injected intramuscularly into the lateral thigh muscle of mice. The injection was given every 2 days. Ten days later, both ovaries of the mice were removed. OVX + Lv‐TRIM27 + ROZ: Ten days after injection of Lv‐TRIM27, both ovaries were resected, and 6 mg/kg of ROZ was administered by gavage for treatment. After 6 weeks of treatment, all the mice were euthanized. Serum and bone tissues were collected for subsequent detection and analysis.

### 2.3. Alizarin Red S Staining

Cells were incubated with osteogenic medium for 7–14 days to allow the formation of opaque calcified nodules. The cell samples were then washed once with phosphate‐buffered saline (PBS), followed by fixation with 4% paraformaldehyde (PFA) for 20 min. Washed nodules were then stained with 0.1% Alizarin Red S (Beyotime, Cat# C0148S, China) solution for 30 min. The stained images were observed and taken using a phase‐contrast microscope (Nikon, Tokyo, Japan). The area stained by Alizarin Red S was quantified using Image J software from over five random fields.

### 2.4. H&E Staining

Bone tissue samples were harvested and fixed in 4% PFA. Decalcification was performed using 10% EDTA for 3 weeks, followed by paraffin embedding. After deparaffinization and rehydration, sections were stained with hematoxylin and eosin reagent. Finally, the observations were conducted under an optical microscope with photographic documentation.

### 2.5. RT‐qPCR

Total RNA in the cells was extracted using TRIzol and reverse transcribed into cDNA using a reverse transcription kit. Then, cDNA reverse transcription and quantitative RT‐PCR were carried out. Quantitative RT‐PCR was performed using Fast SYBR Green PCR Master Mix. The relative expression levels were calculated by the 2^−ΔΔCt^ method, and GAPDH was used as the internal reference gene.

### 2.6. Western Blot

According to the previously reported method [15], a western blot was performed to detect and analyze the protein expression levels of TRIM27, PPARγ, COL1A1, OPN, and OCN in cells and bone tissue.

### 2.7. Alkaline Phosphatase (ALP) Activity Assay

ALP activity was measured using a commercial ALP assay kit according to the manufacturer’s instructions. In brief, cell lysates were collected, centrifuged, and the resulting supernatants were assayed. Absorbance was measured at 405 nm using a spectrophotometer.

### 2.8. Co‐Immunoprecipitation (Co‐IP)

A Co‐IP experiment was conducted according to the previously reported method [16] to analyze the interaction between TRIM27 and PPARγ.

### 2.9. Statistical Analysis

All the data in this study were expressed as mean ± SD. The differences between groups were determined by a paired two‐tailed Student’s test or a two‐way analysis of variance (ANOVA). One‐way ANOVA was used for comparisons among multiple groups. A *p* < 0.05 was considered statistically significant.

## 3. Results

### 3.1. The Expression of TRIM27 in Osteogenic Differentiated Cells and OVX Mice

To investigate the role of TRIM27 in osteoporosis, we first examined the expression of TRIM27 during osteogenic differentiation and in OVX mice. Alizarin Red S staining results (Figure 1(a)) revealed significantly increased calcium deposition in the osteogenic differentiation group. RT‐qPCR analysis (Figure 1(b)) demonstrated significantly elevated mRNA levels of the osteogenic markers COL1A1, OCN, and OPN in osteogenic differentiated cells. Western blot results (Figure 1(c)) confirmed consistent upregulation of these proteins. Furthermore, both RT‐qPCR (Figure 1(d)) and western blot results (Figure 1(e)) showed significantly increased mRNA and protein expression of TRIM27 in the osteogenic differentiation group. In the OVX mice model, H&E staining results (Figure 1(f)) indicated reduced bone surface (BS) and bone volume, alongside increased adipocytes. Moreover, the bone formation makers, OCN and P1NP, were reduced, while the bone resorption maker, CTX‐I, was elevated in OVX mice (Figure 1(g)). As shown in Figures 1(h) and 1(i), OVX mice also exhibited significantly decreased mRNA levels of COL1A1, OCN, and OPN, a trend confirmed at the protein level by western blotting. Consistently, both RT‐qPCR (Figure 1(j)) and western blot (Figure 1(k)) revealed significantly reduced TRIM27 expression in the OVX group.

FIGURE 1The expression of TRIM27 in osteogenic differentiated cells and OVX mice. (a) Osteogenesis was analyzed using the ARS staining assay. RT‐qPCR analysis (b) and western blot (c) were used to detect the mRNA and protein expressions of COL1A1, OPN, and OCN in osteogenic differentiated cells. (d) RT‐qPCR analysis was used to detect the mRNA expression of TRIM27. (e) Representative TRIM27 protein band. (f) Bone formation in mice was detected by H&E staining. (g) Serum OCN, PNP, and CTX‐1 concentrations in sham and OVX mice. RT‐qPCR analysis (h) and western blot (i) were used to detect the mRNA and protein expressions of COL1A1, OPN, and OCN in osteogenic differentiated cells. (j) RT‐qPCR analysis was used to detect the mRNA expression of TRIM27. (k) Representative TRIM27 protein band. ^∗^ *p* < 0.05, ^∗∗^ *p* < 0.01, ^∗∗∗^ *p* < 0.001 vs. the control/sham group, *n* = 3.

**Figure:**
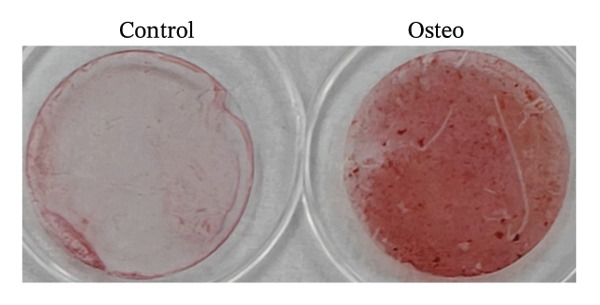
(a)

**Figure:**
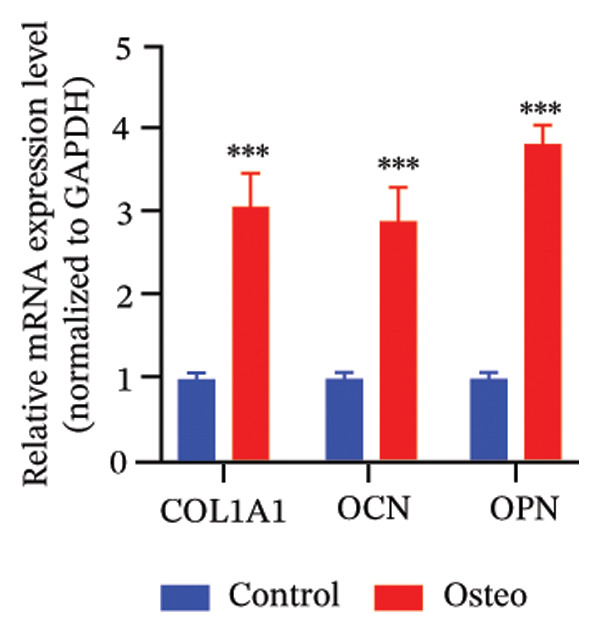
(b)

**Figure:**
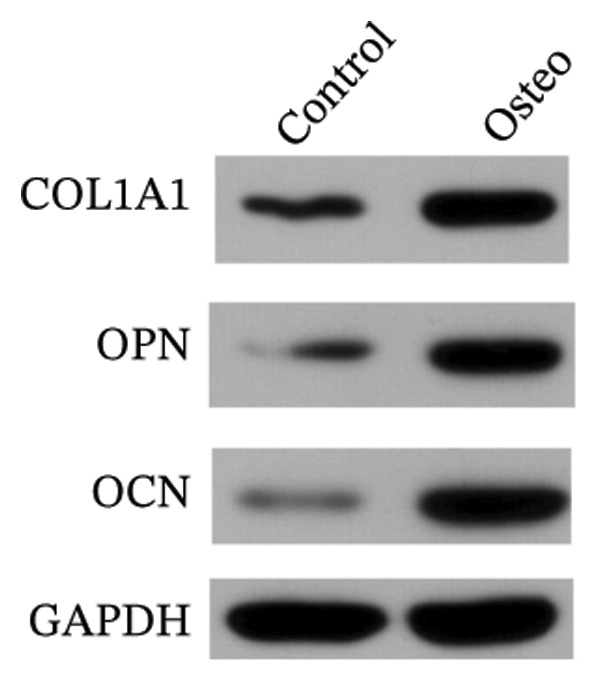
(c)

**Figure:**
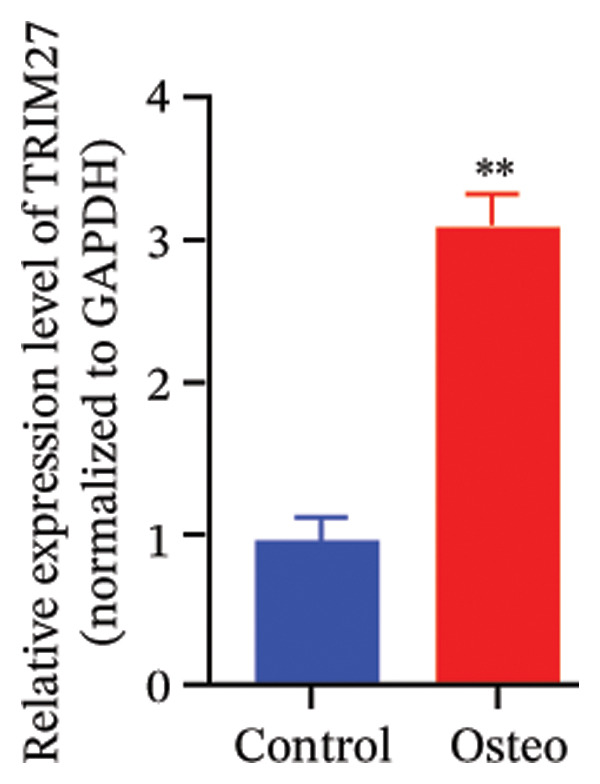
(d)

**Figure:**
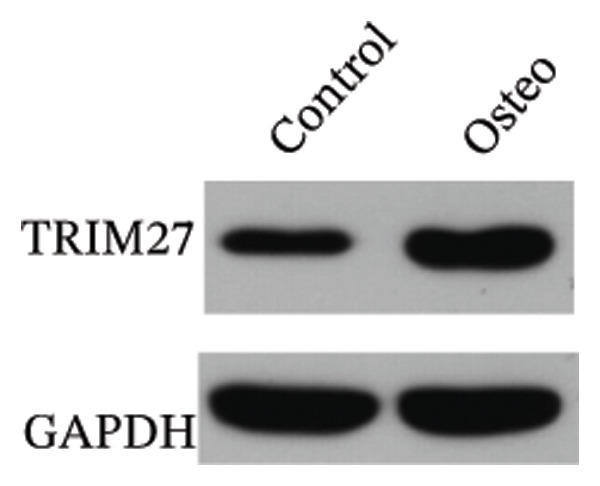
(e)

**Figure:**
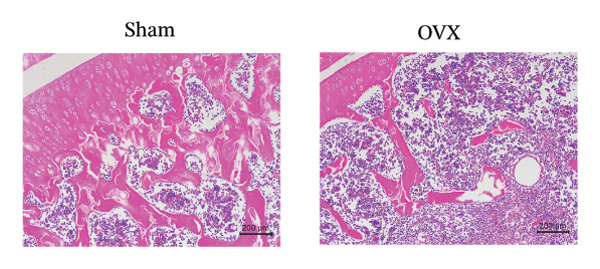
(f)

**Figure:**
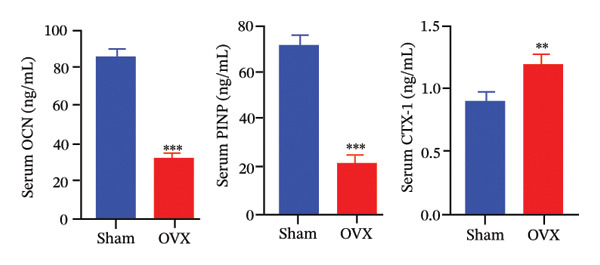
(g)

**Figure:**
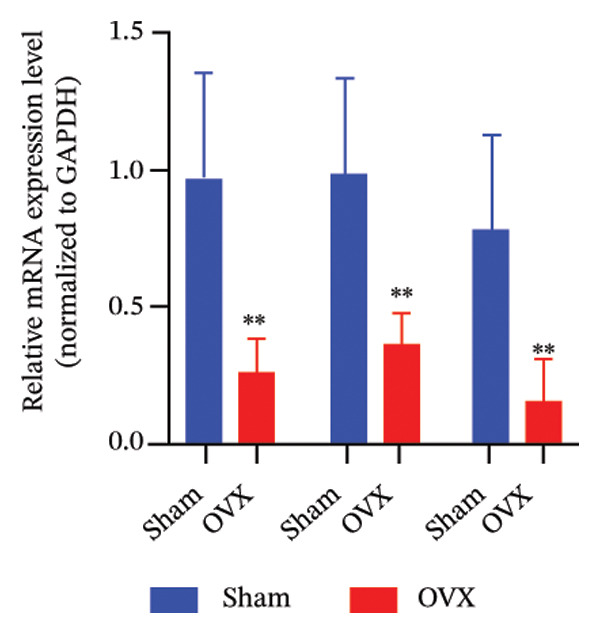
(h)

**Figure:**
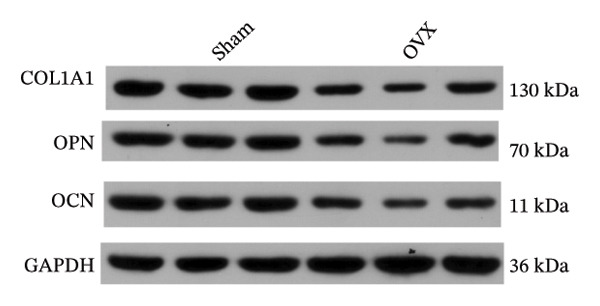
(i)

**Figure:**
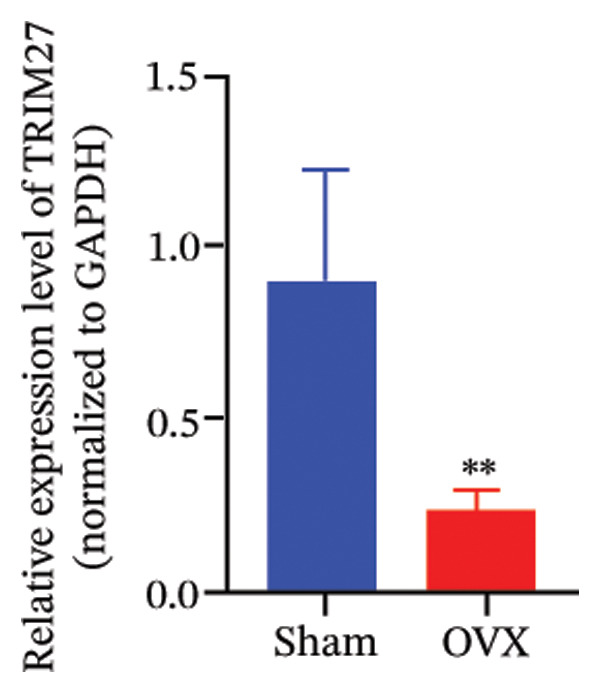
(j)

**Figure:**
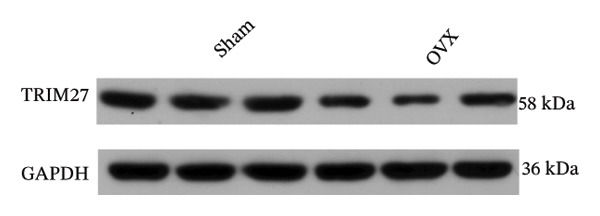
(k)

### 3.2. The Function of TRIM27 in BMSCs Differentiation

To elucidate the role of TRIM27 in the osteogenic differentiation of BMSCs, successful transfection of sh‐TRIM27 (knockdown) and vector‐TRIM27 (overexpression) constructs was first confirmed by RT‐qPCR (Figure 2(a)) and western blot results (Figure 2(b)). As shown in Figure 2(c), after 7 days of osteogenic induction, ALP activity was significantly reduced in the sh‐TRIM27 group compared to the NC group, whereas the vector + TRIM27 group exhibited markedly enhanced ALP activity relative to the vector control group. Alizarin Red S staining results (Figure 2(d)) similarly revealed diminished extracellular matrix mineralization in the sh‐TRIM27 group and augmented mineralization in the vector + TRIM27 group. Subsequent analysis of osteogenic markers demonstrated that mRNA levels of COL1A1, OPN, and OCN were significantly downregulated in the sh‐TRIM27 group compared to the NC group, but robustly upregulated in the vector + TRIM27 group compared to the vector group (Figure 2(e)). Consistent with RT‐qPCR results, western blot results (Figure 2(f)) confirmed identical trends in protein expression for these markers. The findings collectively demonstrated that TRIM27 overexpression promotes osteogenic differentiation in BMSCs.

FIGURE 2The function of TRIM27 in BMSCs differentiation. (a) RT‐qPCR analysis was used to detect the mRNA expression of TRIM27. (b) The representative bands of TRIM27 protein expression. (c) The ALP activities were measured by densitometry at 405 nm. (d) ARS staining to evaluate osteogenic differentiation ability. (e) RT‐qPCR analysis was used to detect the mRNA expression of COL1A1, OPN, and OCN. (f) Western blot was performed to determine the protein expression of COL1A1, OPN, and OCN. ^∗^ *p* < 0.05 and ^∗∗^ *p* < 0.01 vs. the NC group; ^#^ *p* < 0.05 and ^##^ *p* < 0.01 vs. the vector group, n = 3.

**Figure:**
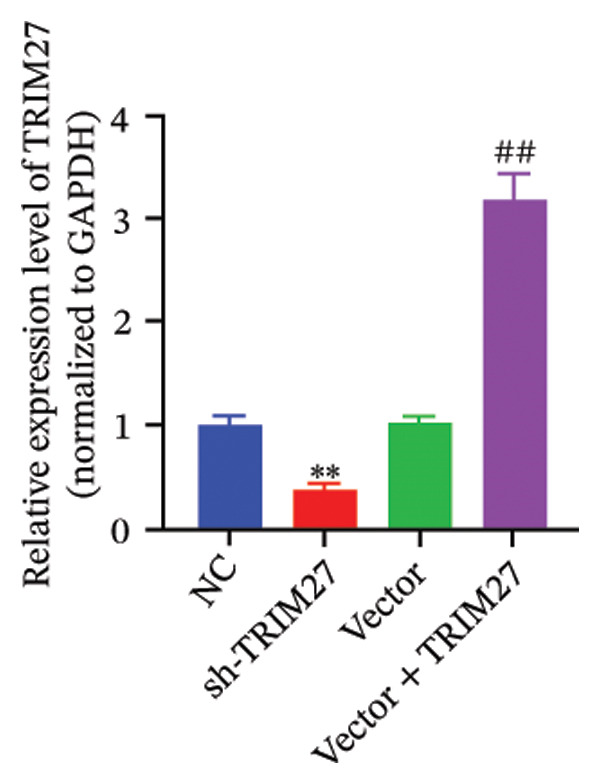
(a)

**Figure:**
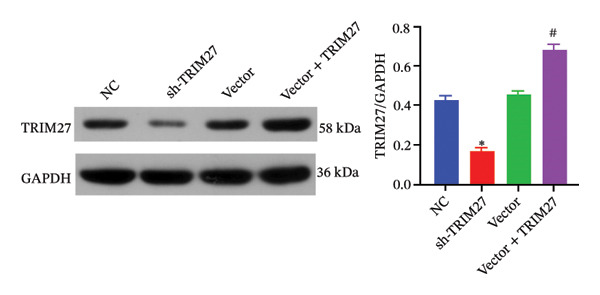
(b)

**Figure:**
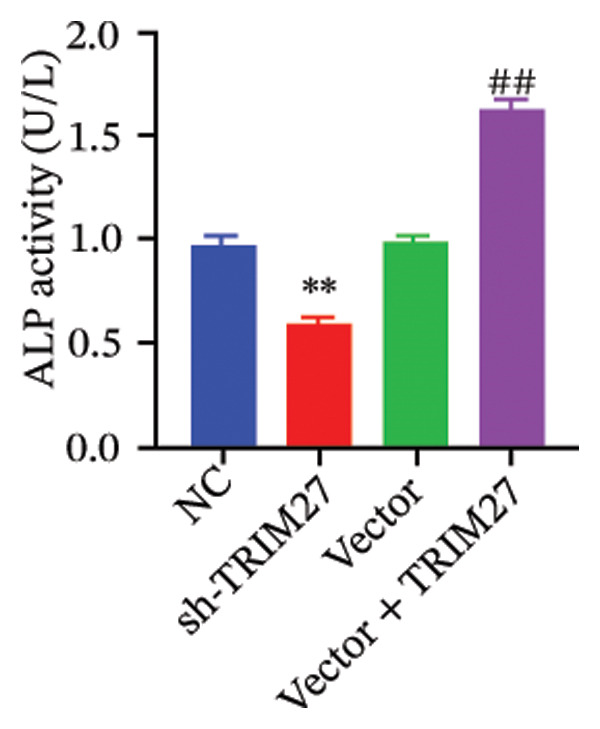
(c)

**Figure:**
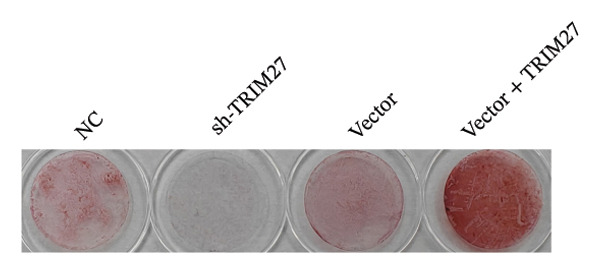
(d)

**Figure:**
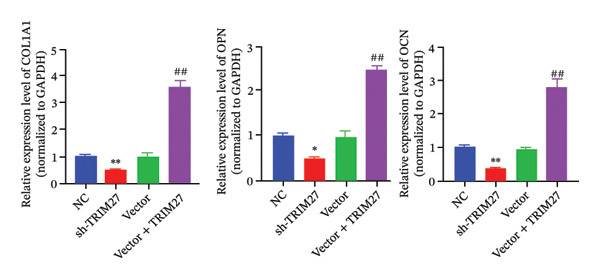
(e)

**Figure:**
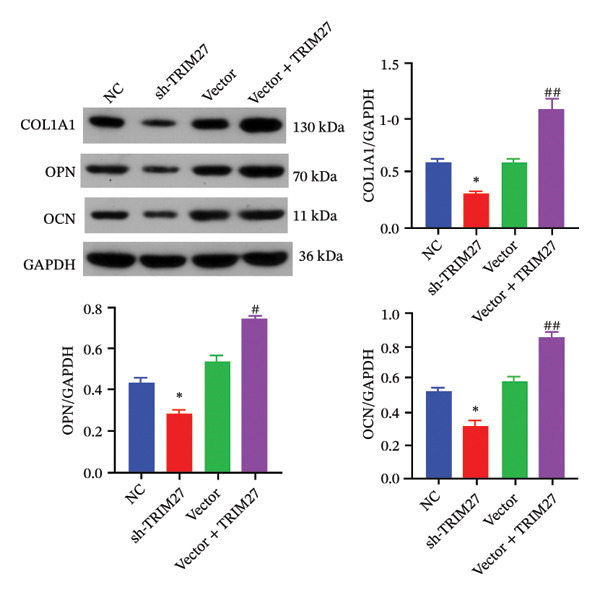
(f)

### 3.3. TRIM27 Regulates PPARγ Protein Degradation via Ubiquitination

TRIM27 is defined as an E3 ubiquitin ligase. Studies have reported that TRIM27 regulates the posttranslational modification of PPARγ to exert physiological effects. Co‐IP experiment results (Figure 3(a)) confirmed the interaction between TRIM27 and PPARγ. Furthermore, overexpression of TRIM27 significantly reduced the level of PPARγ protein, and MG132 could reverse this trend (Figure 3(b)). After treatment with cycloheximide, the protein expression level of PPARγ was analyzed by western blot. It (Figure 3(c)) showed that the degradation of the protein expression level of PPARγ was slowed down in the TRIM27 silencing group. Compared to the control group, the protein degradation of PPARγ in the overexpression TRIM27 group was accelerated. Finally, the western blot results showed that, compared to the NC group, the expression level of PPARγ protein in the sh‐TRIM27 group was significantly upregulated. However, the expression level of PPARγ in the vector + TRIM27 group was significantly decreased compared to the vector group (Figure 3(d)).

FIGURE 3TRIM27 regulates PPARγ protein degradation via ubiquitination. (a) Co‐IP verified the interaction between TRIM27 and PPARγ. (b) The influence of TRIM27 on the ubiquitination of PPARγ. (c) After treatment with cycloheximide, the degradation of PPARγ protein was detected by western blot. (d) Western blot was used to detect the protein expression of PPARγ. ^∗^ *p* < 0.05 and ^∗∗^ *p* < 0.01 vs. the NC group; ^#^ *p* < 0.05 vs. the vector group, *n* = 3.

**Figure:**
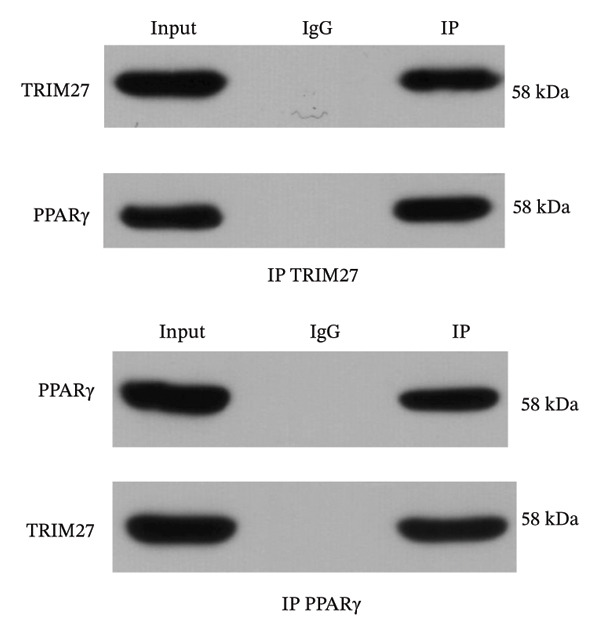
(a)

**Figure:**
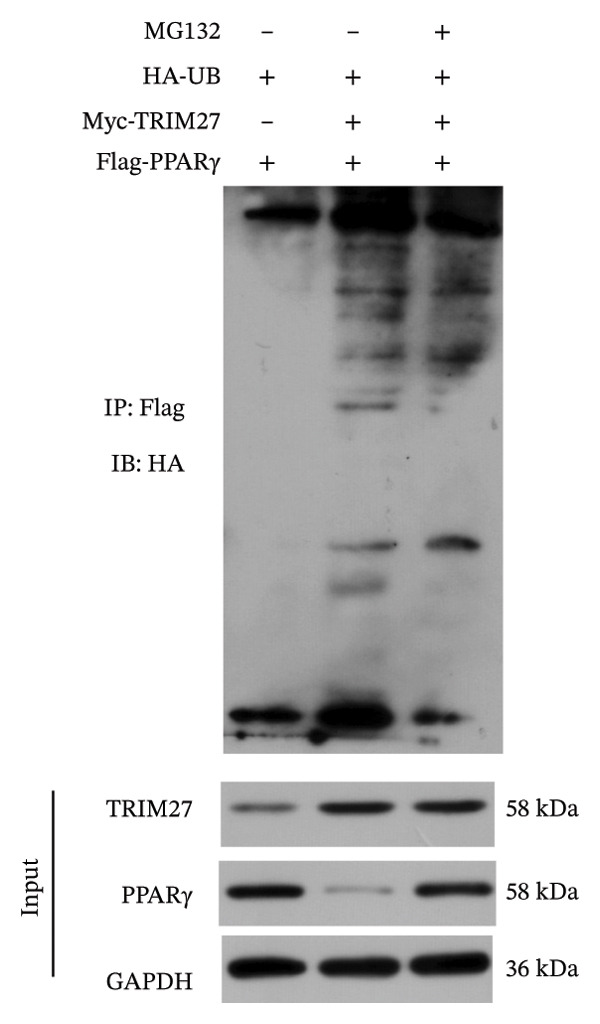
(b)

**Figure:**
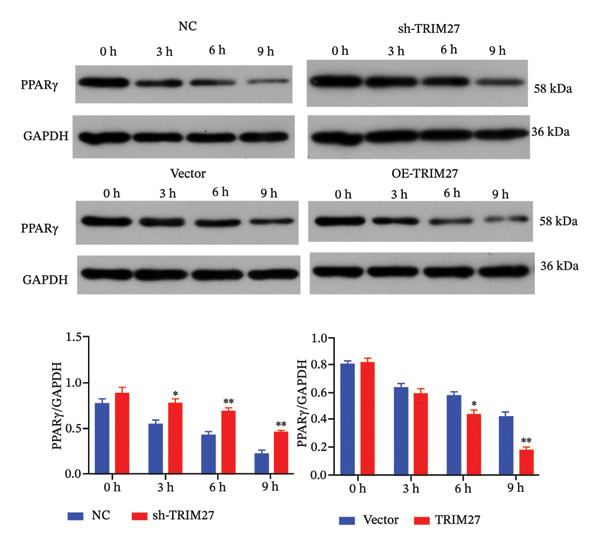
(c)

**Figure:**
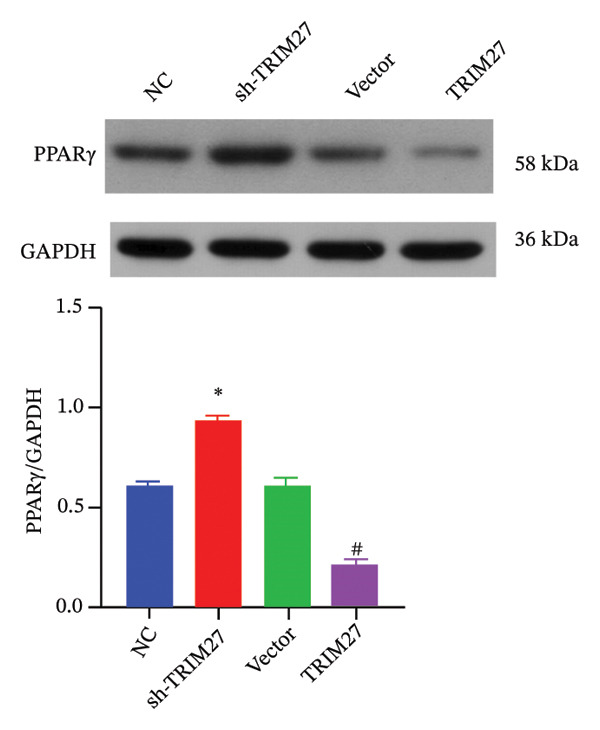
(d)

### 3.4. Knockdown of TRIM27 Suppresses Osteogenic Differentiation by Modulating PPARγ

Next, we transfected sh‐TRIM27 and sh‐PPARγ, respectively, to analyze the molecular mechanism of sh‐TRIM27 on osteogenic differentiation. Western blot results (Figure 4(a)) demonstrated that PPARγ protein expression was significantly decreased in the Osteo + NC group but markedly upregulated upon sh‐TRIM27 transfection. Compared to the Osteo + sh‐TRIM27 group, co‐transfection with sh‐PPARγ (Osteo + sh‐TRIM27 + sh‐PPARγ) substantially reduced PPARγ levels. As shown in Figure 4(b), after 7 days of osteogenic induction, the ALP activity increased significantly in the Osteo + sh‐TRIM27 + sh‐PPARγ group compared to sh‐TRIM27 transfection alone. Alizarin Red S staining (Figure 4(c)) confirmed enhanced extracellular matrix mineralization in the co‐transfection group. RT‐qPCR results (Figure 4(d)) revealed significantly elevated mRNA levels of COL1A1, OPN, and OCN in Osteo + sh‐TRIM27 + sh‐PPARγ compared to the sh‐TRIM27 group, with western blot results (Figure 4(e)) showing concordant protein expression patterns. The above results collectively demonstrated that knockdown of TRIM27 suppresses osteogenic differentiation through PPARγ regulation.

FIGURE 4Knockdown of TRIM27 suppresses osteogenic differentiation by modulating PPARγ. (a) Western blot was used to detect the protein expression of PPARγ. (b) The ALP activities were measured by densitometry at 405 nm. (c) ARS staining to evaluate osteogenic differentiation ability. (d) RT‐qPCR anslysis was used to detect the mRNA expressions of COL1A1, OPN, and OCN. (e) The protein expressions of COL1A1, OPN, and OCN were measured via western blot. ^∗^ *p* < 0.05 and ^∗∗^ *p* < 0.01 vs. the control group; ^#^ *p* < 0.05 and ^##^ *p* < 0.01 vs. the Osteo + NC group; ^&^ *p* < 0.05 and ^&&^ *p* < 0.01 vs. the Osteo + sh‐TRIM27 group, n = 3.

**Figure:**
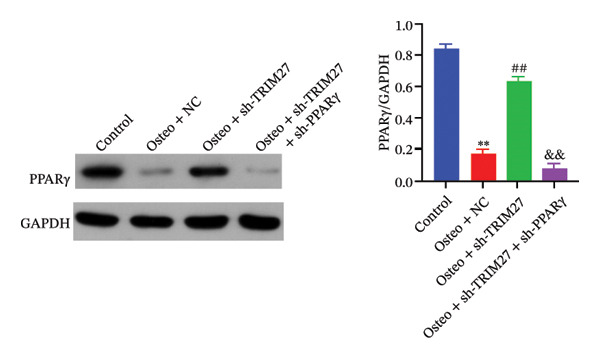
(a)

**Figure:**
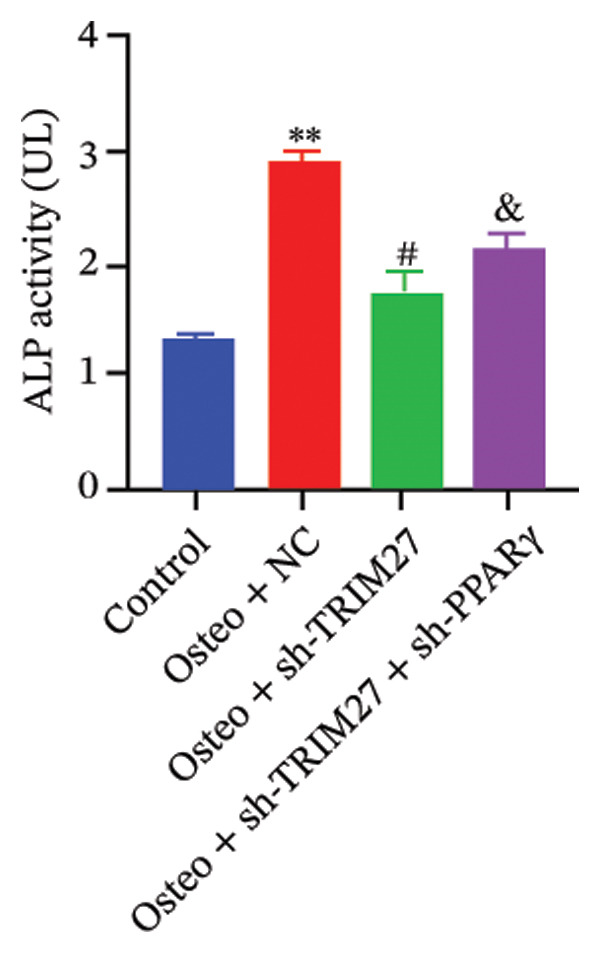
(b)

**Figure:**
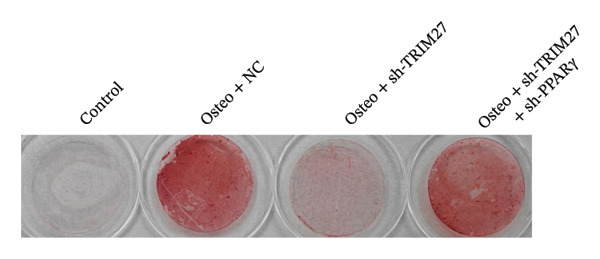
(c)

**Figure:**
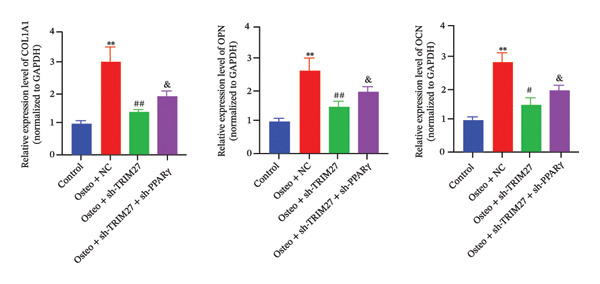
(d)

**Figure:**
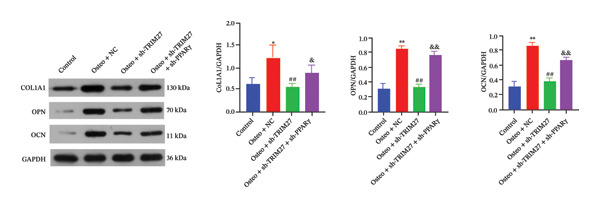
(e)

### 3.5. TRIM27 Improves Osteoporosis of OVX Mice by Modulating PPARγ

We conducted a study on the regulatory molecular mechanism of TRIM27 on osteoporosis in OVX mice. Western blot results (Figure 5(a)) revealed significantly elevated PPARγ protein levels in OVX mice compared to controls. This increase was reversed by TRIM27 knockdown, which markedly reduced PPARγ expression. Treatment with the PPARγ agonist ROZ in sh‐TRIM27 mice (OVX + sh‐TRIM27 + ROZ) restored PPARγ protein levels compared to the OVX + sh‐TRIM27 group. H&E staining (Figure 5(b)) demonstrated that OVX mice overexpressing TRIM27 (Lv‐TRIM27) and treated with ROZ (OVX + Lv‐TRIM27 + ROZ) exhibited reduced BS, decreased bone volume, and increased adipocyte formation relative to the OVX + Lv‐TRIM27 group. Consistent with this phenotype, RT‐qPCR results (Figure 5(c)) showed significantly decreased mRNA expression of osteogenic markers (COL1A1, OPN, and OCN) in the OVX + Lv‐TRIM27 + ROZ group. Western blot results (Figure 5(d)) confirmed the corresponding downregulation of these marker proteins. Collectively, it demonstrated that TRIM27 ameliorates osteoporosis by suppressing PPARγ.

FIGURE 5TRIM27 improves osteoporosis of OVX mice by modulating PPARγ. (a) The representative bands of PPARγ expression. (b) The representative H&E staining images of the distal femur sections. (c) RT‐qPCR analysis was used to detect the mRNA expressions of COL1A1, OPN, and OCN. (d) Western blot was performed to detect the protein expressions of COL1A1, OPN, and OCN. ^∗^ *p* < 0.05 and ^∗∗^ *p* < 0.01 vs. the sham group; ^#^ *p* < 0.05 and ^##^ *p* < 0.01 vs. the OVX group; ^&^ *p* < 0.05 and ^&&^ *p* < 0.01, vs. the OVX + Lv‐TRIM27 group, *n* = 3.

**Figure:**
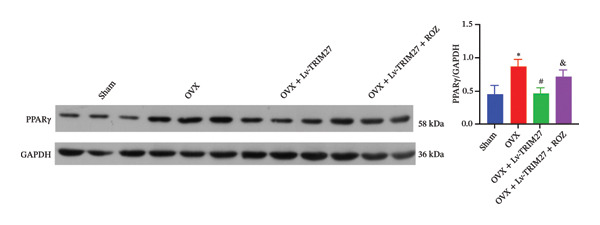
(a)

**Figure:**
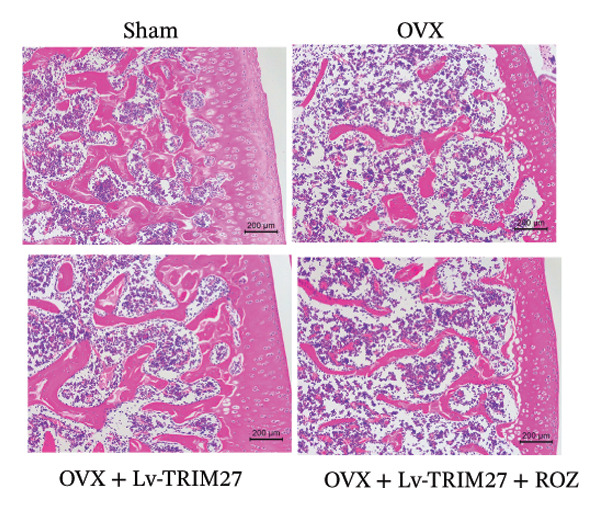
(b)

**Figure:**
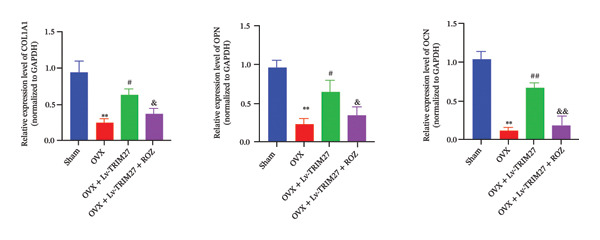
(c)

**Figure:**
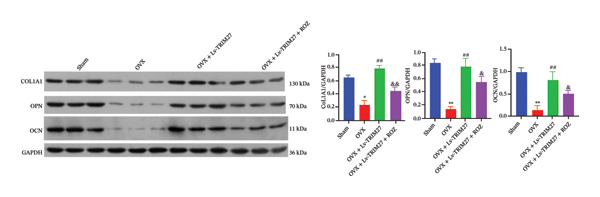
(d)

## 4. Discussion

Osteoporosis is fundamentally characterized by the disruption of bone remodeling homeostasis, leading to progressive bone loss and increased skeletal fragility. The key risk factors that induce osteoporosis include postmenopausal estrogen decline, aging, nutritional deficiencies (particularly calcium/vitamin D), and detrimental lifestyle habits. BMSCs, the precursors of osteoblasts, possess multipotent differentiation capabilities [17]. Recent studies [18, 19] highlighted that promoting BMSC‐specific osteogenic differentiation plays a pivotal role in osteoporosis therapy. In this study, we found that TRIM27 plays an important role in the osteogenic differentiation of BMSC and the disease development process of osteoporotic mice. The results indicated that the expression of TRIM27 was positively correlated with the osteogenic differentiation ability of BMSCs. Conversely, its downregulation in OVX mice would exacerbate the osteoporosis phenotype, and overexpression of TRIM27 could alleviate the development of osteoporosis. Therefore, our research provides theoretical support for TRIM27 as a potential target for the treatment of osteoporosis.

Previous studies [20, 21] implicated the TRIM family in bone homeostasis regulation. Exosomes derived from BMSCs upregulate the expression of TRIM25. TRIM25 promotes the ubiquitination and degradation of TREM1, regulates the polarization of M2 macrophages, and thereby promotes osteogenic differentiation [22]. The absence of TRIM21 coordinates the coupling of osteoblast and osteoclast through the YAP1/β‐catenin signaling, delaying bone loss induced by OVX and LPS [23]. AFPR inhibited ROS and NFATc1 by targeting TRIM38‐mediated degradation of TRAF6 proteasome, slowed down bone loss, and mitigated the development of premature ovarian failure [24]. In line with these reports, we observed that TRIM27 overexpression increased mRNA and protein levels of osteogenic markers (COL1A1, OPN, and OCN) in BMSCs, supporting a proosteogenic role for TRIM27 in vitro.

Mechanistically, our data support a model in which TRIM27 promotes reduction of PPARγ protein levels in a proteasome‐sensitive manner. This discovery provides a molecular explanation for the inverse relationship between the expressions of TRIM27 and PPARγ observed during osteogenesis. To further verify this regulatory axis, we conducted rescue experiments using the PPARγ agonist ROZ. The results confirmed that the osteogenic effect of TRIM27 was mediated by inhibiting the activity of PPARγ, thereby establishing a clear TRIM27–PPARγ regulatory pathway in bone formation. Consistent with previous reports [25, 26], it has been indicated that PPARγ agonists (ROZ) can overactivate PPARγ and exacerbate the risk of osteoporosis. On the contrary, isopsoralen can inhibit PPARγ phosphorylation, regulate the PI3K/AKT/mTOR pathway, reverse osteogenic inhibition, and alleviate the development of osteoporosis.

Our study reveals that overexpression of TRIM27 can alleviate osteoporosis by rebalancing the PPARγ‐mediated shift from osteogenesis to adipogenesis. This study identifies TRIM27 as a positive regulator that can counteract the PPARγ‐driven adipogenic shift in osteoporosis. This contrasts with TRIM27’s role as a negative regulator of NF‐κB signaling in osteoclastogenesis, highlighting the multifaceted functions of TRIM27 in bone physiology. Second, it establishes PPARγ ubiquitination as a previously unrecognized regulatory mechanism in bone metabolism, expanding the established transcriptional control mechanisms. Limitations of the present study include the incomplete biochemical mapping of ubiquitination events described above and the need for expanded in vivo phenotyping (for example, quantitative micro‐CT, histomorphometry, and serum bone turnover markers) to further substantiate the protective effects of TRIM27 in OVX models. Future studies should also examine whether TRIM27 cooperates with or functions independently of other bone‐relevant E3 ligases, and whether modulation of TRIM27 has beneficial effects in additional models and in human samples.

In summary, this study not only clarifies the key role of TRIM27 in osteogenic differentiation and osteoporosis but also opens up new avenues for therapeutic development. Our research provides a molecular mechanism of the TRIM27–PPARγ regulatory axis, laying the foundation for innovative approaches to combat osteoporosis.

## Author Contributions

Liya Zhang and Junfeng Li supervised the project and formulated the concept and experimental design. Liya Zhang performed the experiments. Liya Zhang and Benjuan Wu assisted with experiments and provided the materials. Benjuan Wu prepared the original manuscript. Liya Zhang wrote the main manuscript. All authors reviewed the manuscript.

## Funding

No funding was received for this study.

## Ethics Statement

Animal experiments were approved by the Animal Ethics Committee of Tianjin First Central Hospital.

## Consent

The authors have nothing to report.

## Conflicts of Interest

The authors declare no conflicts of interest.

## Data Availability

Data are available on request from the authors.
